# Skeletal and dentoalveolar bilateral dimensions in unilateral palatally impacted canine using cone beam computed tomography

**DOI:** 10.1186/s40510-017-0160-6

**Published:** 2017-02-20

**Authors:** Mariel Franchesca D´ Oleo-Aracena, Luis Ernesto Arriola-Guillén, Yalil Augusto Rodríguez-Cárdenas, Gustavo Armando Ruíz-Mora

**Affiliations:** 1 School of Dentistry, Universidad Científica del Sur, Lima, Perú; 2 Division of Radiology and Periodontics, Faculty of Dentistry, Universidad Iberoamericana–UNIBE, Santo Domingo, Dominican Republic; 3 Division of Orthodontics and Division of Oral and Maxillofacial Radiology, School of Dentistry, Universidad Científica del Sur, Lima, Perú; 4 Division of Oral and Maxillofacial Radiology, School of Dentistry, Universidad Científica del Sur, Lima, Perú; 5 Division of Orthodontics, Faculty of Dentistry, Universidad Nacional de Bogotá, Bogotá, Colombia; 6 Calle Los Girasoles # 194, Dpto. # 302, Urb. Residencial Los Ingenieros de Valle Hermoso, Santiago de Surco, Lima, Perú

**Keywords:** Canines impacted, Unilateral impaction, Skeletal dimensions

## Abstract

**Background:**

The aim of this investigation was to compare skeletal and dentoalveolar measurements of subject with unilateral palatally impacted canine versus the unaffected contralateral side on cone beam computed tomography (CBCT).

**Methods:**

A cross-sectional study (split mouth design) that included 28 CBCTs (i.e., 56 sides) with unilaterally impacted maxillary canines was performed. After conducting a pilot test to gauge the researcher, heights and widths of skeletal and dentoalveolar variables obtained in the maxilla were measured using coronal and axial views. The angulations of incisors were also measured, and the side with impaction and the unaffected side were compared. Paired sample *t* test and Wilcoxon signed-rank test were used.

**Results:**

Significant statistical differences (2 mm, *p* < 0.001) were found between the impacted and non-impacted side measurements from the mid-palatine raphe to the first premolar (proximal alveolar bone crest between the canine (deciduous or permanent) and first premolar); the distance were significantly lower (12.72 ± 2.25 mm) than in the side without impaction (14.67 ± 2.00 mm). Also, the central and lateral incisor angulations showed significant reductions; presenting disto-angulated incisors on the impacted canine side (86.14 ± 7.70° and 74.75 ± 12.67°, respectively) and mesial-angulated incisors on the non-impacted side (91.63 ± 6.79° and 81.21 ± 8.56° respectively). The other skeletal and dentoalveolar measurements showed no significant differences.

**Conclusions:**

The width from the median raphe to the first premolar is lower in the side of maxillary palatal impacted canines than in the side without impaction. Lateral angulations of incisors were disto-angulated on the side of impacted canines. Both conditions have clinical implications in the orthodontic treatment.

## Background

In pathological terms, an impacted tooth can be defined as an abnormal state in which the tooth is completely or partially covered by mucoperiosteum and bone, distant from the site and time that it should be erupted in the oral cavity [1–3]. Impacted canine in the palatal position occurs 3 to 6 times more often than buccal position [4, 5]. Impacted canines are twice as common in women as in men, and the incidence in the maxilla is more than double compared to the jaw [6]. Unerupted canines are the second most common group suffering impaction surpassed only by impacted third molars, its reported prevalence varies from 0.2% to 2.8% [2, 3]. Two main theories have been proposed to explain the emergence of palatal impacted maxillary canines: the “orientation” and “genetic” theories [7]. The lack of space in the dental arch can prolong retention of deciduous canines. Adjacent lateral incisors absence, root dilaceration, and ankyloses of the permanent canines are the most common local factors associated with maxillary impacted canines [8–10].

Investigations pointed out a lack of the accurate characterization of alveolar bone dimensions and the environment in the affected area [11, 12]. The impaction can lead to reduced bone dimensions, or affect dental angulations of the nearby teeth. There are a few studies [13, 14] comparing specifically the impacted area with the area that had adequate canine eruption in the same individual. These results indicate the consequences generated by the impaction of a canine. Kanavakis et al. [14] concluded that the root of lateral incisors adjacent to palatal impacted canines is angulated more mesially compared to that of lateral incisors adjacent to normally erupted canines.

With the advent of cone beam computed tomography (CBCT), three-dimensional representations (3D) of the teeth and bone are presented in high resolution. Tadinada et al. [13] reported that alveolar bone dimensions (buccal-palatal width and height of the nasal floor to the alveolar ridge) and the perimeter of the arch are significantly reduced in the impacted side when compared with the non-impacted side. However, they did not evaluate lateral angulations of the long axis of incisors, neither the nasal cavity width nor lateral basal width, which also could be affected. It has been stated that maxillary transverse discrepancies increase the possibility of impacted canines [15]. According to Becker et al. [16], three-dimensional and unilateral precise determination of the position of impacted canines is important for the clinician to determine the prognosis of an aligned tooth on the dental arch.

The literature [13, 14] has little information about how the morphology and maxillary dimensions can affect the eruption and subsequent impaction of maxillary canines. For these reasons, the aim of this investigation was to compare skeletal and dentoalveolar dimensions in a sample with unilateral palatally impacted canines versus the unaffected side. Analyzing the characteristics of these dimensions and determining how they influence the impacted canines on vertical and transverse measurements using coronal and axial views on CBCT have been little reported in the scientific literature.

## Methods

This retrospective and cross-sectional study (including a split mouth design) was approved by the Ethics Committee of Científica del Sur University, Lima, Peru, with the No. of approval 000258.

The sample consisted of CBCTs with unilateral palatally impacted canines. CBCTs of subjects attended in “Imaging Diagnostic Center CDI” Lima, Peru, from January 2010 to December 2015 were included. The sample size calculation required 25 sides with and 25 sides without impacted maxillary canine. We calculated this sample considering a mean difference of 6° in the lateral incisor angulation as a clinically relevant difference between sides with and without impacted canine. A standard deviation of 8.58° was considered (obtained from a preliminary pilot study) with a two-sided significance level of 0.05 and a power of 80%. Although a minimum of 25 sides were required, we included 28 sides with and 28 sides without impacted maxillary canine (in overall 56 sides). This amount was selected from a sample of 960 CBCTs.

The inclusion criteria for images selection were CBCTs of children or adults over 15 years old of both sexes, periodontally healthy, and with canines fully calcified, including unilateral palatally impacted canines located in the maxillary [17]. We included impacted canines located in sector 2 (if the cusp tip of the canine is between the major axes of the lateral and central) and sector 3 (if the cusp tip of the canine is between the major axis of the lateral and the first premolar) as rates by Erikson and Kurol [17]. Both groups were included as one in the sample. We include cases with deciduous canines in occlusion on the affected side to avoid a lack of arch development.

Exclusion criteria were subjects with previous orthodontic treatment, dento-maxillary traumas, maxillary canine transpositions, agenesis, craniofacial malformations, odontogenic pathologies, and CBCTs including impacted maxillary bilateral canines and bucally impacted canines.

### Measurements

CBCT scans of all patients were obtained using Vatech E-woo model Picasso Master 3D scanner (Vatech, Hwaseong, South Korea) set to 8 mA, 90 Kv with a flat panel 25 × 20 cm, 30 × 30 cm. The 20 × 19 cm field of view producing 0.3 mm isotropic voxel sizes and exposure time of 20 s was used for acquiring the image volume.

DICOM images were analyzed on RealScan 2.0 software, using multiplanar reconstructions as well as the evaluation using 3D reconstructions in rendering volume and anteroposterior radiographs derived of the CBCTs, in a Samsung Intel Core i7-4770 workstation, displayed on a S19C150 LCD Samsung monitor with LED backlit of 18.5-inch, widescreen, with a resolution of 1366 × 768 pixels in a dimly lit room. All assessments were made by one calibrated radiologist, and the measurements were expressed in millimeters (mm) and degrees (°). The calibration was performed by a specialist in Oral and Maxillofacial Radiology. Intra-examiner calibration was performed. The intra-class correlation coefficient was performed, with 0.90 (confidence interval to 95% 0.801–0.995) of agreement accepted to proceed with the research. Ten scans per day were evaluated.

**Fig. 1:**
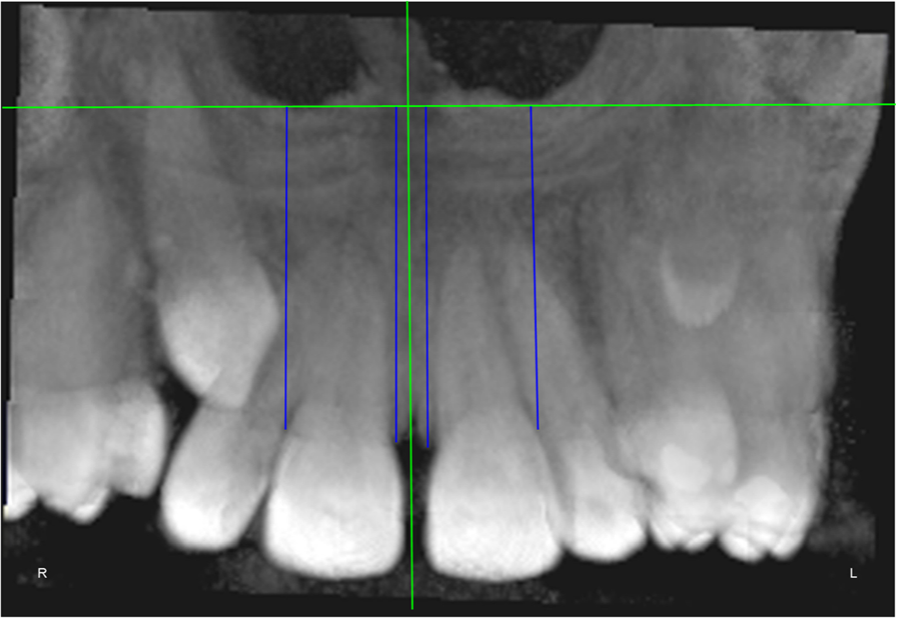
Anterior alveolar ridge height. Measured in millimeters from the bony ridge of upper incisors by drawing a straight line parallel to the midsagittal plane till the floor of the nostrils on the side of the impacted canine and the side without impaction

**Fig. 2:**
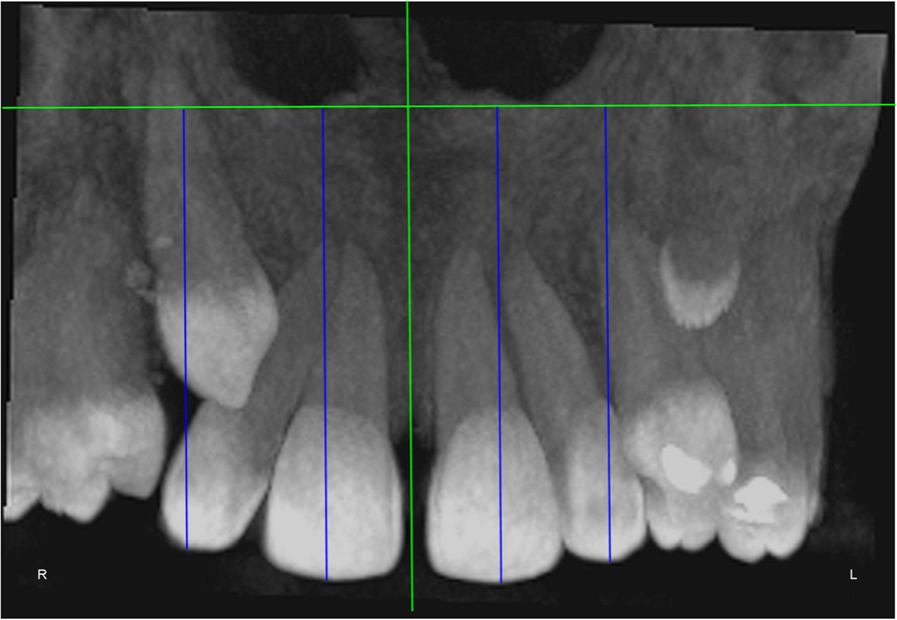
Anterior dentoalveolar height. Measured in millimeters from the incisal edge of upper incisors by drawing a straight line parallel to the midsagittal plane till the floor of the nostrils on the side of the impacted canine and the side without impaction

**Fig. 3:**
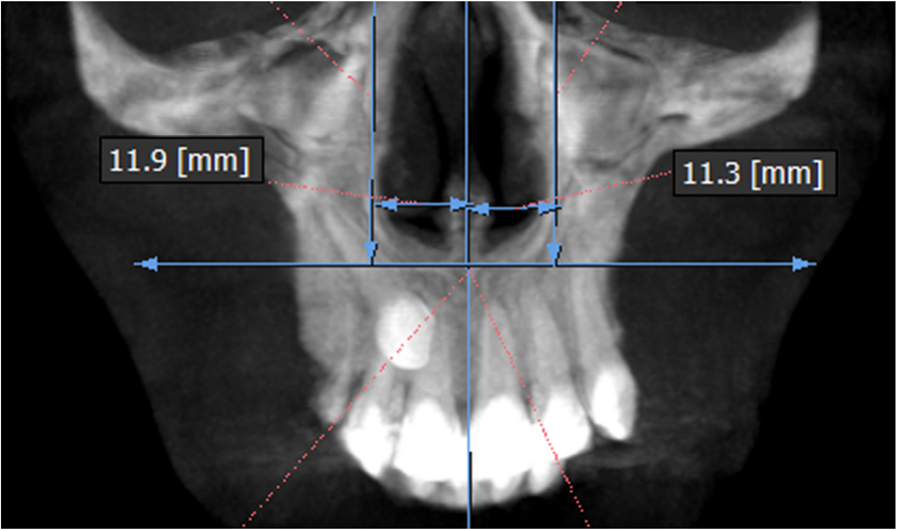
Nasal cavity width. Measured in millimeters from the anterior nasal spine to the lateral wall of the nasal base on the side of the impacted canine and canine without impaction

**Fig. 4:**
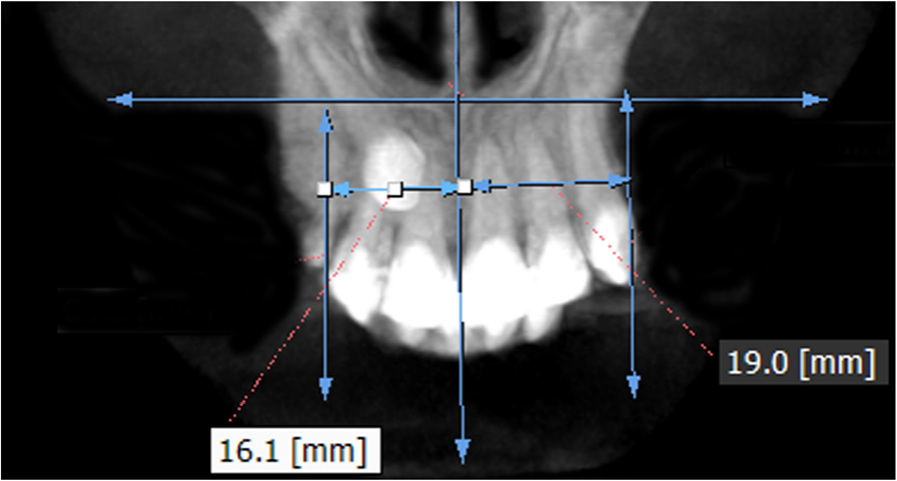
Basal lateral width. Measured in millimeters from the anterior nasal spine to the dentoalveolar outermost rim on the side of the impacted canine and canine without impaction

**Fig. 5:**
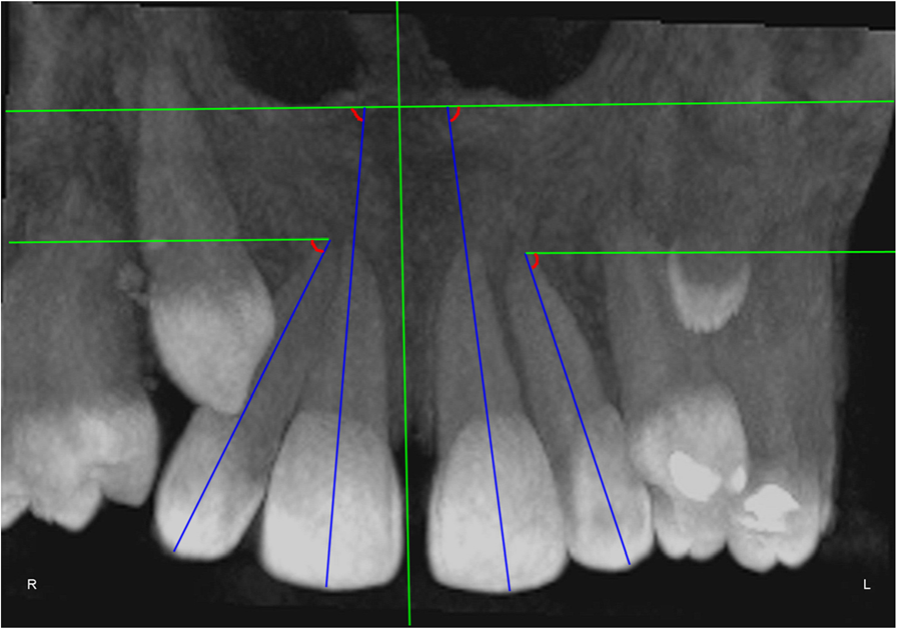
Lateral angulation of long axis of the incisors with respect to the nasal horizontal plane. Value of the external angle of the longitudinal axis of the central and lateral incisors of both quadrants with respect to the tangent of the nostril floor

**Fig. 6:**
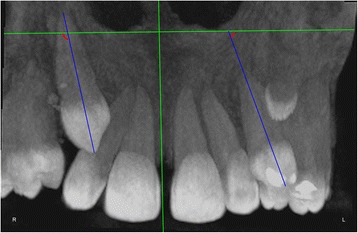
Lateral angulation of long axis of canines with respect to the nasal horizontal plane. Value of the external angle of the longitudinal axis of the impacted canine and of that with no impaction with respect to the tangent of the nostril floor

**Fig. 7:**
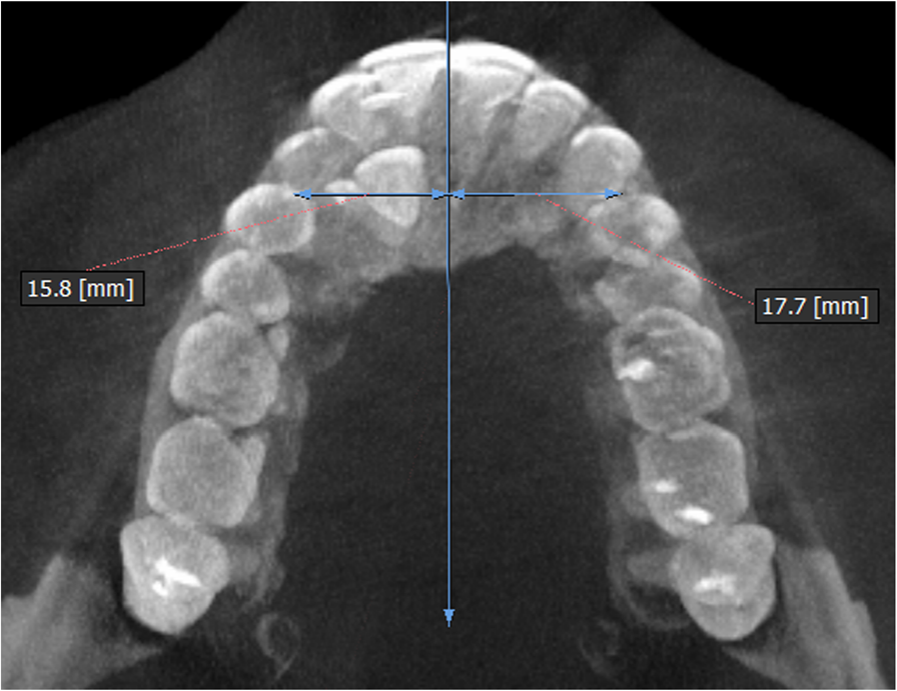
Premolar width. Distance in millimeters from the middle palatine raphe till proximal alveolar bone crest between the canine and first premolar on each side; measured in the axial cut at bone crest level

The measurements were performed on anteroposterior radiographs derived from the CBCTs in maximum intensity projection (MIP coronal views) and axial sections of CBCTs (Table 1, Figs. 1, 2, 3, 4, 5, 6, and 7), similar to the measurements shown in the study of Yan et al. [18]; the heights of skeletal and dent alveolar variables and angulations of incisors and canines were obtained in anteroposterior radiographs derived from the CBCTs comparing the impacted side with the unaffected side, and the widths of dent alveolar variables were measured in axial sections (Table 1)

**Table 1:** Skeletal and dentoalveolar measurements

Variables	Definition
Anterior alveolar ridge height	Measured in millimeters from the bony ridge of upper incisors by drawing a straight line parallel to the midsagittal plane till the floor of the nostrils on the side of impacted canine and side without impaction (Fig. 1)
Anterior dentoalveolar height	Measured in millimeters from the incisal edge of upper incisors by drawing a straight line parallel to the midsagittal plane till floor of the nostrils on side of impacted canine and side without impaction (Fig. 2)
Nasal cavity width	Measured in millimeters from the anterior nasal spine to the lateral wall of the nasal base on the side of impacted canine and the canine without impaction (Fig. 3)
Basal lateral width	Measured in millimeters from the anterior nasal spine to the outermost dentoalveolar rim on the side of impacted canine and the canine without impaction (Fig. 4)
Lateral angulation of long axis of the incisors with respect to the nasal horizontal plane	Value of the external angle of the longitudinal axis of the central and lateral incisors of both quadrants with respect to the tangent of the nostril floor (Fig. 5)
Lateral angulation of long axis of canines with respect to the nasal horizontal plane	Value of the external angle of the longitudinal axis of the impacted canine and which has no impaction with respect to the tangent of the nostril floor (Fig. 6)
Premolar width	Distance in millimeters from the middle palatine raphe to proximal alveolar bone crest between the canine (deciduous or permanent) and first premolar on each side, measured in the axial cut at bone crest level (Fig. 7)

### Statistical analysis

Statistical analysis was performed using the SPSS version 22.0 (Chicago III). Descriptive statistics was performed (group of unilateral impacted canine compared with the contralateral side without impaction). The Shapiro-Wilk test was assessed to determine whether the data had normal distribution; when there was normality, results were compared using paired sample *t* test, and when there was no normality, the Wilcoxon signed-rank test was performed. Statistical significance was established at a level of *p* < 0.05 for all tests.

## Results

The male sex showed a population of 11 patients (39.29%) and 17 female patients (60.71%). The mean age of male patients was 22.09 ± 4.70 years and for female patients 23.12 ± 5.17 years (Table 2).

**Table 2:** Demographic characteristics and canine angulations according to the side of impaction

Sex	*n* (%)	Age mean (SD)	Canine angulations	Mean	SD
Male	11 (39.29)	22.09 (4.70)	Impacted	120.53°	27.85°
Female	17 (60.71)	23.12 (5.17)	Non-impacted	85.02°	9.99°
Total	28 (100)				

There are not significant differences found when the anterior alveolar ridge height and anterior dentoalveolar height were measured, *p* > 0.05(Table 3).

**Table 3:** Comparison of heights of skeletal and dentoalveolar variables obtained in the maxilla depending on the condition side of impaction

Measured variables	Canine condition	*N*	Mean	Standard deviation	Min	Max	Variance	*P*
Anterior dentoalveolar height of central incisor	Impacted	28	29.72	4.51	22.30	37.80	20.34	0.948^a^
	Non-impacted	28	29.65	4.46	22.90	37.80	19.89	
Anterior dentoalveolar height of lateral incisor	Impacted	28	27.81	3.68	21.10	35.00	13.57	0.713^a^
	Non-impacted	28	28.17	3.68	22.80	35.30	13.60	
Anterior alveolar ridge height of central incisor	Impacted	28	21.10	3.91	15.20	27.70	15.32	0.861^a^
	Non-impacted	28	20.92	4.00	15.00	28.10	16.05	
Anterior alveolar ridge height of lateral incisor	Impacted	28	20.56	3.74	13.50	27.00	14.00	0.713^a^
	Non-impacted	28	20.20	3.71	13.20	27.70	13.76	

^a^Paired sample *t* test

For the dentoalveolar variable of width from premolar to the mid-palatine raphe, the impacted condition average was 12.72 ± 2.25 mm and the condition without impaction, 14.67 ± 2.00 mm. The value of statistical significance obtained through Wilcoxon signed-rank test was *p* < 0.001, significant differences were observed (Table 4).

**Table 4:** Comparison of widths in bone and dentoalveolar dimensions of sample depending on the condition side of impaction

Measured variables	Canine condition	*n*	Mean	Standard deviation	Min	Max	Variance	*p*
Nasal cavity width	Impacted	28	12.17	1.84	8.40	15.50	3.39	0.634^a^
	Non-impacted	28	12.41	1.95	9.30	17.20	3.82	
Basal lateral width	Impacted	28	19.01	2.55	13.40	23.50	6.53	0.298^a^
	Non-impacted	28	19.69	2.29	14.90	24.40	5.24	
Premolar width to median raphe	Impacted	28	12.72	2.25	10.20	20.10	5.08	**<**0.001^b^
	Non-impacted	28	14.67	2.00	11.70	18.40	4.03	

^a^Paired sample *t* test

^b^Wilcoxon signed-rank test

Table 5 shows comparisons of variables from lateral angulations of long axis of incisors with respect to the nasal horizontal plane; for the lateral angulation of central incisors, the impacted condition average was 86.14° ± 7.70° and the condition without impaction, 91.63° ± 6.79°. The value of statistical significance obtained through the Wilcoxon signed-rank test was *p* = 0.008; significant differences were observed. For the lateral angulation of lateral incisors, the impacted condition average was 74.75° ± 12.67° and the condition without impaction, 81.21° ± 8.56°. The value of statistical significance obtained through paired sample *t* test was *p* = 0.030; significant differences were observed.

**Table 5:** Comparison of lateral angulation of long axis of incisors with respect to the nasal horizontal plane in bone and dentoalveolar dimensions of sample depending on the condition side of impaction.

Measured variables	Canine condition	*n*	Mean	Standard deviation	Min	Max	Variance	*p*
Central incisor angulation	Impacted	28	86.14	7.70	58.20	103.00	59.41	0.008^a^
	Non-impacted	28	91.63	6.79	76.50	105.00	46.18	
Lateral incisor angulation	Impacted	28	74.75	12.67	45.00	99.60	160.61	0.030^b^
	Non-impacted	28	81.21	8.56	68.70	102.00	73.42	

^a^Wilcoxon signed-rank test

^b^Paired sample *t* test

## Discussion

Unilateral palatally impacted maxillary canines represent an asymmetric dentoalveolar and/or basal bone structure of the right or left anterior segment of the maxillae [17]. The main objective of this study was to compare the skeletal and dentoalveolar dimensions of the maxillae in a sample with unilaterally impacted canines versus the contralateral unaffected side. There are a few studies [13, 14] with similar methodology but did not use CBCT or did not include all variables like this study; also, coronal and axial views have been little reported in the scientific literature.

In order to avoid measurement and sample selection bias, a pilot study was performed to ensure the reliability of the results. Furthermore, the intra-observer concordance was almost perfect for bone measurements and dentoalveolar dimensions of the sample, ensuring the reliability of the measurements.

An important limitation to be considered was the relatively small sample size of this study, but this was due to the selection of CBCTs only in patients with unilateral palatally impacted canines, with a prevalence that ranges from a minimum of 0.92% to a maximum of 4.3% [2, 3]. We found this required sample from a total of 960 CBCTs (28 CBCTs, 2.94% of prevalence), although this prevalence of impacted canines in the sample did not depict the prevalence in the overall population because our sample was formed by CBCTs of patients that assisted to one radiological images center seeking orthodontic or surgical diagnosis.

In this study, the most prevalent gender was females, confirming that the impacted upper canines are produced twice as common in women than in men, with a ratio of 2 or 3 to 1 [1–3]. One main reason because the women were more prevalent in this sample added to etiological factor is probably the mere fact that women are esthetically more oriented to get orthodontic treatment.

When comparing the bone heights and dentoalveolar dimensions of the sample according to the condition of the impacted canine side, no statistically significant differences were found. The study of Tadinada et al. [13] showed different results, the alveolar bone dimensions (bucco-palatal width and length of the nasal floor to the alveolar ridge) and the maxillary arch perimeter were significantly lower on the impacted side, compared to that on the not impacted side. However, we think that the incisor heights should not affect because the sequence of eruption of incisors is prior to canines. In our study, all subjects had Latin American origin, and great variability related to the crown size was not expected; finally, we did not find difference into these heights between both sides (with and without impaction).

Jacoby [6] reported that 85% of palatal impacted canines were in patients with an adequate perimeter arc. Similarly, Stellzig et al. [19] reported that there was enough perimeter of the arc in 82% of palatal impacted canines. However, in our study, significant differences were observed on the measurements from the mid-palatine raphe to the first premolar since the affected side was significantly lower than the non-impacted side. This was because the side of the impacted canine have not been sufficiently developed, compared with the unaffected side where canines have normally eruptions. In our sample, we found all deciduous canines in occlusion is possible that the lack of permanent canine eruption can affect the inter-canine distance and the transversal measurements; however, more future studies can compare these measures in cases with or without persistence of deciduous canine because the lack of tooth mass could account for a lack of arch development. Similar results were found by Tadinada et al. [13]; the length reduction of the arc on the affected side may also be due to the lack of eruption of the impacted permanent canine.

The clinical significance of our findings with respect to treatment implies a greater attention to correct the transverse asymmetries mainly at level of the first premolar on the side that includes an impacted canine. The severity of this asymmetry (approximately 2 mm between both sides) should be corrected only with dental alignment; however, in cases of greater asymmetry including unilateral cross bite, the asymmetric expansions should be taken into account [20].

Likewise, statistically significant differences were observed when the lateral angulations of the long axis of incisors were compared according to the side of the impacted canine. The lateral angulation of the long axis of the incisors was lower on the impacted side presenting disto-angulated incisors on the side of impacted canine and mesial-angulated on the non-impacted side.

Meanwhile, the lateral angulation of the long axis of the canines showed greater angulations on the impacted side compared to the non-impacted side with mesial tipping in the impacted canine. This was similarly presented in the study of Hanke et al. [21] where the inclinations and lengths of vectors for impacted canines were higher (mesial tipping) than in those non-impacted canines. The inclinations of the long axis of the canines in relation to the three reference planes are particularly suitable for comparisons, and in their study, significant differences were detected (*p* < 0.001). Similarly, this was also reported by Kanavakis et al. [14], where the crown-root angulation of lateral incisors adjacent to palatal impacted canines differ compared to that of lateral incisors adjacent to normally erupted canine, but this study was made on the panoramic radiographs, where the long axis of the root of the lateral incisors adjacent to palatal impacted canines form a more mesial angle to the crown (approximately 2.5°), when compared to the lateral incisors adjacent to normally erupted canines.

The present study included impacted canines located in sectors 2 and 3 as rated by Ericson and Kurol [17]; we did not included sector 1 because this condition is less frequent than the other two and the effect on the angulations of incisor is more expected, and ideally, we should form three groups according to this condition, but due to the small sample managed in the study, we did not classified the sample into these groups. It is recommended the use of this classification on future research. Other recommendation for future studies is the use of 3D analytic techniques for evaluation of shape differences between both sides with or without canine impaction, and this would provide other information about bone contours and bone volume; specifically, in this paper we attempt to compare measurements easily recognized by orthodontists and with clinical value, mainly in the coronal and axial views.

Probably, the orthodontic treatment in unilateral palatally impacted canine requires its previous traction; the alignment of the incisors without distancing the impacted canine could expose the roots of the incisors with the impacted canine due to their distal angulation with respect to the opposite side without impaction. Furthermore, the orthodontists should have a greater attention to correct the transverse asymmetries mainly at level of the width from median raphe to first premolar on the affected side with an impacted canine.

In conclusion, three measurements on the side of impacted canines were significantly lower than on the side without impaction; width from premolar to the mid-palatine raphe and lateral angulations of incisors (lateral and central) showed significant reduction, presenting disto-angulated incisors on the side of impacted canine.

## Conclusions

The orthodontic treatment of unilateral maxillary impacted canine should correct the transverse asymmetry mainly at level of the premolar width on the affected side with respect to the not impacted side and prevent the contact of the root of the incisors with impacted canines due to the disto-angulation of the lateral and central incisors on the affected side.

## Abbreviations

CBCT: Cone beam computed tomography.
